# Antioxidant Effect of *Lippia alba* (Miller) N. E. Brown

**DOI:** 10.3390/antiox2040194

**Published:** 2013-09-26

**Authors:** Claire E. Chies, Cátia S. Branco, Gustavo Scola, Fabiana Agostini, Adriana E. Gower, Mirian Salvador

**Affiliations:** Institute of Biotechnology, University of Caxias do Sul, Caxias do Sul, RS 95070560, Brazil; E-Mails: cechies@yahoo.com.br (C.E.C.); csbranc1@ucs.br (C.S.B.); gustavo.scola@gmail.com (G.S.); fagosti1@ucs.br (F.A.); aegower@ucs.br (A.E.G.)

**Keywords:** antioxidant, flavonoids, radical scavenger, *Lippia alba*

## Abstract

*Lippia alba* is a shrub found in all regions of Brazil and other countries in South and Central America. *L. alba* exhibits variability among its different accessions, showing differences in morphology and in the composition of its essential oil. This study evaluated the phenolic profiles and the antioxidant activities of seven different accessions of *L. alba*. The seven accessions of *L. alba* studied exhibited an important phenolic content, and all accessions demonstrated antioxidant activity with different efficacies. The main flavonoids in all accessions were apigenin, luteolin, naringin and rutin. The Santa Vitória do Palmar accession exhibited higher naringin and total phenolic content. This extract was able to reduce hydrogen peroxide-induced oxidative damage in tissue homogenates of cerebellum, cerebral cortex, hippocampus and liver of Wistar rats.

## 1. Introduction

The species *Lippia alba* (Miller) N. E. Brown, is known in Brazil as “erva-cidreira”. *L. alba* is an aromatic shrub in the Verbenaceae family that grows wild in Central and South America. This plant is used in folk medicine as a sedative [1]; a treatment for cardiovascular diseases, diabetes [2] and hepatic diseases [3]; a syrup against bronchitis [4]; and an anticandida agent [5]. *L. alba* expresses morphological and phytochemical variations, which characterize the different accessions of this plant [6]. High qualitative and quantitative variations of the essential oil composition of *L. alba* accessions have been reported previously [6,7,8]. This plant also contains phenolic compounds, primarily flavonoids [9] that exert antioxidant properties. However, possible variations in the levels of these compounds and/or the antioxidant activity of different *L. alba* accessions are not known. Phenolic compounds possess many biological effects, including the scavenging of free radicals, which minimizes the incidence of several diseases that are associated with oxidative damage, such as found in Alzheimer’s and Parkinson’s diseases, cirrhosis, cancer and atherosclerosis [10].

Different methods to measure the antioxidant activity of natural compounds have been developed, and more than one method should be used to produce more reliable results. Among the *in vitro* assays, the ability of one compound to donate electrons to the stable radical 2,2-diphenyl-1-picrylhydrazyl (DPPH^•^) is one of the most used and reproducible assays [11]. Moreover, the ability of some compounds to act in a similar manner as the antioxidant enzymes superoxide dismutase (SOD) and catalase (CAT) is also commonly used [12,13]. Assays using mammalian living cells are useful in the identification of antioxidant activity [14,15,16]. Brain cells are highly vulnerable to oxidative damage due to their high consumption of oxygen, the presence of large amounts of easily oxidizable polyunsaturated fatty acids, and an abundance of redox-active transition metal ions [17]. Oxidative damage is also associated with liver diseases, including cirrhosis and tumors [18]. Brain and hepatic cells were chosen for this study because *L. alba* is used in folk medicine as a sedative [1] and for the treatment of hepatic diseases [3]. 

Few studies have investigated the *in vitro* antioxidant activity of *L. alba* extracts obtained with organic solvents [19,20] and essential oils [21]. However, the differences in phenolic content and the antioxidant activity of different accessions of *L. alba* are not known. In this perspective, this work compared total phenolic content, main flavonoids levels and the antioxidant activity of seven different accessions of *L. alba*. The ability of the highest phenolic content extract to reduce the oxidative damage in tissue homogenates of central nervous system (CNS) and liver of Wistar rats was also evaluated.

## 2. Experimental Section

### 2.1. Plant Material

*L. alba* (Miller) N. E. Brown plants were collected from six different localities (Barra do Quaraí, Caxias do Sul, Harmonia, Pelotas, Porto Alegre and Santa Vitória do Palmar) in the state of Rio Grande do Sul in southern Brazil. One plant, the Pinheira accession, was collected in the state of Santa Catarina, which is also in southern Brazil (Table 1).

**Table 1:** Localization of the geographical regions of origin of the different *Lippia alba* accessions.

City	State	Country	Latitude	Longitude	Altitude (m)
Barra do Quaraí	RS	Brazil	30°23′14.3″	56°27′20″	35
Caxias do Sul	RS	Brazil	29°09′78″	51°08′65.9″	760
Harmonia	RS	Brazil	31°19′19.01″	52º17′17.02″	126
Pelotas	RS	Brazil	31°44′29.23″	52°20′43.76″	13.24
Pinheira	SC	Brazil	27°53′3.96″	48°35′4.15″	3
Porto Alegre	RS	Brazil	30°4′10.58″	51°8′27.83″	47
Santa Vitória do Palmar	RS	Brazil	33°31′4.36″	53°22′9.55″	24

The accessions were maintained in a greenhouse at the Institute of Biotechnology, University of Caxias do Sul, Caxias do Sul (29°10′122″ S 51°11′118″ W), Brazil. Plants were propagated by cutting the branches and maintaining the descendants, which were genetically identical to the mother plant. Voucher specimens were identified by the herbarium of the University of Caxias do Sul, Rio Grande do Sul, Brazil (Barra do Quaraí HUCS26630; Caxias do Sul HUCS24689; Harmonia HUCS35972; Pelotas HUCS26631; Pinheira HUCS35971; Porto Alegre HUCS26632; Santa Vitória do Palmar HUCS32197).

### 2.2. Preparation of *Lippia alba* Extracts

Plant leaves were collected on the morning of April 2009 and dried at a constant temperature of 32 °C. The dried leaf powders (10 g) were extracted with 200 mL of hexane, chloroform and ethanol, consecutively, using a Soxhlet apparatus. The last extracts (ethanolic) were filtered and concentrated in a rotary vacuum evaporator to completely remove the solvent. These extracts were dissolved in 10 mL ethanol, and the solutions were used for determination of total phenolic content, main flavonoids and antioxidant activity. The effects of *L. alba* on rat tissues were evaluated using the extract with the highest naringin and total phenolic content, Santa Vitória do Palmar, which was dissolved in 2.5 mL dimethylsulfoxide plus 7.5 mL distilled water. This solution was dissolved in water to obtain a final concentration of 10 μg/mL. All solutions were prepared immediately prior to use.

### 2.3. Determination of Total Phenolic Content

Total phenolic content of *L. alba* extracts was measured using the Singleton and Rossi modification of the Folin–Ciocalteau colorimetric method [22]. Briefly, 200 μL of the extracts were assayed in 1000 μL Folin–Ciocalteau reagent and 800 μL sodium carbonate (7.5%, w/v). The mixture was vortexed and diluted (1:10) in distilled water. The absorbance was measured at 765 nm (SP-220 spectrophotometer, Biospectro/Brazil) after 30 min, and the total phenolic content is expressed as equivalents of gallic acid/ dry leaves (mg/g).

### 2.4. Quantification of the Main Flavonoids of *Lippia alba*

A hydrolysis of the ethanol extracts was performed to release the aglycones of the flavonoid glycosides. The extracts were diluted in ethanol to a final volume of 50 mL. Twenty-five milliliters of this solution were added to 0.5 mL of an aqueous solution of hexamethylenetetramine 0.5% (w/v) followed by 30 mL acetone and 1 mL hydrochloric acid 37% (w/v). This mixture was refluxed for 30 min and filtered at room temperature. Twenty milliliters of this solution was diluted in distilled water (30 mL) and extracted with ethyl acetate (15 mL). This procedure was repeated three times. The ethyl acetate fraction was concentrated in a rotary evaporator and resuspended in 20 mL methanol.

HPLC analysis evaluated apigenin, catechin, epicatequin, kaempferol, luteolin, naringin, quercetin, rutin, and taxifolin content using a series HP1100 equipped with a UV detector and quaternary pumping system as described previously [23]. Flavonoid separation was performed in a 5 µm Lichrospher RP18 column, 250 mm 4 mm. The hydrolyzed extracts were eluted at 1 mL/min (20 µL injection volume) using a binary solvent system as the mobile phase. Solvent A was methanol, and solvent B was a water/acetic acid glacial mixture (100/1 v/v). The following gradient conditions were used: solvent A 5% and solvent B 95% (0 min), solvent A 40% and solvent B 60% (25 min), solvent A 60% and solvent B 40% (40 min), solvent A 90% and solvent B 10% (50 min), solvent A 90% and solvent B 10% (55 min), solvent A 5% and solvent B 95% (60 min). Flavonoids were monitored by UV absorbance at 350 nm. All chromatographic procedures were performed at 25 °C. The quantities of the main flavonoids were estimated from the standard curves of standard compounds (all from Sigma-Aldrich). Flavonoids concentrations are expressed as µg/g of dry leaves.

### 2.5. Antioxidant Activity of the Different Accessions of *Lippia alba*

Antioxidant activity of the seven extracts of *L. alba* was measured using DPPH^•^ (2,2-diphenyl-1-picrylhydrazyl) radical scavenging ability, and superoxide dismutase-*like* and catalase-*like* assays. The DPPH^•^ assay was performed using a modified Yamaguchi *et al.* [11] method. Briefly, the ethanolic stock solutions of the extracts were diluted to different concentrations (0.05% to 0.50%, v/v) using ethanol p.a. These solutions were added to a Tris-HCl buffer (100 mM, pH 7.0) containing 250 µmol L^−1^ DPPH^•^ dissolved in ethanol. Tubes were stored on dark for 20 min, and the absorbance was measured at 517 nm. The results are expressed as IC_50_ (the amount of the extract needed to scavenger 50% of DPPH^•^). Superoxide dismutase-*like* assay was measured as the inhibition of the self-catalytic adrenochrome formation rate at 480 nm in a reaction medium containing 1 mmol L^−1^ adrenaline (pH 2.0) and 50 mmol L^−1^ glycine (pH 10.2). This reaction was performed at 30 °C for 3 min [24]. The results are expressed as IC_50_ (µL of extract needed to reduce 50% of the adrenochrome formation). Catalase-*like* assay determined the hydrogen peroxide decomposition rate at 240 nm [25]. The results are expressed in mmol of H_2_O_2_ decomposed/min.

### 2.6. Effects of *Lippia alba* Extract in Brain and Liver Tissues of Wistar Rats

The extract of *L. alba* with the highest naringin and total phenolic content (Santa Vitória do Palmar) was also studied in brain and livers tissue homogenates of thirty-day-old Wistar rats. The rats were obtained from the breeding colony of the Federal University of Rio Grande do Sul, and maintained at approximately 25 °C on a 12-h light/dark cycle with food and water available *ad libitum*. All experiments were conducted in accordance with the NIH Guide for the Care and Use of Laboratory Animals. All efforts were made to minimize animal suffering and the number of animals necessary to produce reliable scientific data.

Ten male rats were euthanized by decapitation without anesthesia, and the brain and liver were rapidly excised on an iced Petri dish. The cerebellum, cerebral cortex, hippocampus, and liver were dissected. Tissues from all rats were mixed and homogenized in PBS (pH 7.4) using a ground glass-type Potter–Elvehjem homogenizer. The homogenates were centrifuged (800× *g*) for 10 min at 4 °C, the pellets were discarded, and the supernatants were used immediately. Aliquots (1000 µL) were added to the *L. alba* extracts for 30 min, and 5 mmol L^−1^ H_2_O_2_ was added. Tubes were maintained for 1 h at 37 °C under constant agitation. Samples including only the extract or H_2_O_2_ (5 mmol L^−1^) were also evaluated.

Lipid and protein oxidative damage, and activity of the antioxidant enzymes, superoxide dismutase (EC1.15.1.1) and catalase (EC1.11.1.6) were measured. Lipid peroxidation was monitored using the formation of thiobarbituric acid reactive species (TBARS) during an acid-heating reaction. This method has been widely adopted as a sensitive method for the measurement of lipid peroxidation. Trichloroacetic acid (1000 μL, 5%) was added to 250 μL of the samples, and the solution was centrifuged at 7000× *g* for 10 min. Sulfuric acid (1000 μL, 3 mol L^−1^) was mixed with thiobarbituric acid (1000 μL) solution. The reaction mixture was incubated in a boiling water bath for 15 min and cooled to room temperature. *N*-butanol (3500 μL) was added and centrifuged at 7000× *g* for 5 min. The absorbance was read at 532 nm [26]. The results are expressed as nmol of TBARS/mg of protein. Oxidative damage to proteins was determined by carbonyl groups based on the reaction with dinitrophenylhydrazine (DNPH). DNPH reacts with protein carbonyls to form hydrazones that are measured spectrophotometrically. DNPH (200 µL, 10 mmol L^−1^), or HCl (200 μL, 2 mol L^−1^) for control, were added to the supernatants (50 μL). The reaction mixture was incubated in the dark for 30 min with agitation. Trichloroacetic acid (250 μL, 20%) was added, and the solution was centrifuged at 4000× *g* for 8 min. The supernatant was discarded, and the pellet was washed 3 times with ethanol-ethyl acetate (1:1) to remove free reagents. The samples were centrifuged, and the pellets were dissolved in a guanidine solution (600 μL, 6 mol L^−1^) at 37 °C for 15 min. Absorbance was read at 365 nm, and results are expressed as nmol of DNPH/mg of protein [27]. Superoxide dismutase activity was measured spectrophotometrically as the inhibition of the ratio of autocatalytic adrenochrome formation at 480 nm in a reaction medium containing 1 nmol L^−1^ adrenaline (pH 2.0) and 50 nmol L^−1^ glycine (pH 10.2). This reaction was performed at a constant temperature of 30 °C for 3 min. The results are expressed as superoxide dismutase units/mg of protein. One unit of SOD is defined as the amount of enzyme that inhibits the rate of adrenochrome formation by 50% [24]. Catalase activity was measured according to the method described by Aebi [25]. The assay determines the rate of H_2_O_2_ decomposition at 240 nm. The reaction was conducted at 30 °C for 1 min. The results are expressed as units/mg of protein. One unit is defined as the amount of enzyme that decomposes 1 mmol of H_2_O_2_ in 1 min at pH 7.4. Total protein was determined as described by Bradford [28] using bovine serum albumin as standard. All absorbances were measured in spectrophotometer model UV-1700 (Shimadzu, Kyoto, Japan).

### 2.7. Statistical Analysis

All measurements were performed in triplicate, and the means and standard deviation (S.D.) are reported. Data underwent an analysis of variance (ANOVA) followed by Tukey’s post hoc test and Pearson correlation using SPSS version 16.0 (SPSS, Chicago, IL, USA). Values of *P* ≤ 0.05 were considered significant.

## 3. Results and Discussion

### 3.1. Total Phenolic and Flavonoid Content in the Extracts

The different accessions of *L. alba* exhibited different profiles of phenolic compounds. The Harmonia accession exhibited the lowest level of total phenolic content, and the highest total phenolic content was observed in the Santa Vitória do Palmar accession. HPLC analysis demonstrated presence of the flavonoids apigenin, luteolin, naringin and rutin, in all seven different accessions of *L. alba*. Naringin exhibited the highest concentration in all accessions. The Caxias do Sul accession presented the highest levels of apigenin (73.36 ± 3.30 µg/g of dry leaves). The Harmonia accession contained the highest levels of luteolin (67.82 ± 3.53 µg/g of dry leaves) and rutin (34.53 ± 2.13 µg/g of dry leaves). The Santa Vitória do Palmar accession exhibited the highest concentrations of naringin (707.04 ± 7.67 µg/g of dry leaves) (Table 2). Catechin, epicatechin, kaempferol, quercetin and taxifolin were not identified in any accession. Apigenin, luteolin [9] and biflavonoids [29] have been observed in leaves of *L. alba* previously. However, this study is the first to detect the presence of naringin and rutin in this plant. No biflavonoids were observed in the extracts in our work, which may be due to the different extraction methods used and/or differences in the plants that were evaluated.

The seven accessions were acclimated in a greenhouse in Caxias do Sul, Brazil. Therefore, the differences in the phenolic compound profiles were likely not attributable to differences in climate, soil, incidence of light, altitude, latitude, planting method, fertilization or cultural practice. It is more probable that these accessions are different cultivars of *L. alba*. In fact, notable morphological differences (data not shown) between the leaves of the different accessions were observed, which corroborates this hypothesis.

**Table 2:** Concentration of the main flavonoids (µg/g of dry leaves) and total phenolic content (equivalents of gallic acid/dry leaves; mg/g) in the different accessions of *Lippia alba*.

Accessions of *Lippia alba*	Apigenin	Luteolin	Naringin	Rutin	Total phenolic content
Barra do Quaraí	67.08 ± 0.82 ^b^	13.08 ± 0.99 ^f^	133.92± 1.06 ^d^^,e^	22.82 ± 0.37 ^b^	338.39 ± 4.00 ^c^
Caxias do Sul	73.36 ± 3.30 ^a^	24.81 ± 0.84 ^c^^,d^	116.56 ± 4.09 ^e^	21.93 ± 0.55 ^b^	289.73 ± 2.78 ^e^
Harmonia	58.58 ± 2.90 ^c^	67.82 ± 3.53 ^a^	436.65 ± 5.78 ^b^	34.53 ± 2.13 ^a^	226.15 ± 4.63 ^f^
Pelotas	39.30 ± 0.95 ^d^	20.75 ± 1.15 ^d^^,e^	155.19 ± 6.44 ^c^	22.59 ± 0.19 ^b^	364.01 ± 1.85 ^b^
Pinheira	28.71 ± 1.61 ^e^	27.03 ± 0.79 ^c^	144.42 ± 4.35 ^c^^,d^	24.40 ± 1.40 ^b^	320.80 ± 4.45 ^d^
Porto Alegre	15.57 ± 0.94 ^f^	16.95 ± 0.49 ^e^^,f^	136.12 ± 0.95 ^c^^,d^	24.14 ± 0.22 ^b^	368.95 ± 4.32 ^b^
Santa Vitória do Palmar	38.19 ± 0.62 ^d^	35.72 ± 0.66 ^b^	707.04 ± 7.67 ^a^	24.74 ± 0.12 ^b^	403.51 ± 4.32 ^a^

Different letters correspond to values significantly different by analysis of variance (ANOVA) and Tukey’s post hoc test for *p* ≤ 0.05 for each flavonoid or total phenolic content.

### 3.2. Antioxidant Activity of the Different Accessions of *Lippia alba*

DPPH^•^ scavenging ability is a widely used method for the evaluation of antioxidant activities in a relatively short time compared to other methods [30], and is very useful for the screening of a large number of samples [13]. Our study demonstrated that all accessions of *L. alba* exerted important antioxidant activity in this assay, with Harmonia being the most efficient. The antioxidant activity (DPPH^•^ assay) of *L. alba* hydroalcoholic [20] and methanolic [19] extracts from the leaves and flowers of *L. alba* has been reported, and this corroborates the data in this work. All accessions of *L. alba* also demonstrated SOD- and CAT-*like* activities (Table 3). Pinheira has the higher SOD-like activity, and Barra do Quaraí the higher CAT-like activity. SOD catalyzes the dismutation of a superoxide anion (O_2_^−•^) to oxygen and hydrogen peroxide (H_2_O_2_), and CAT converts H_2_O_2_ to water and molecular oxygen [31]. The ability of the extracts to behave as antioxidant enzymes plays a pivotal role in maintenance of the physiological redox equilibrium, avoiding or decreasing the oxidative stress. Positive correlations between the phenolic compounds and the antioxidant activity of *L. alba* accessions were observed (Table 4), which suggests that these compounds are at least partially responsible for the antioxidant activity observed in this study. Phenolic compounds scavenge superoxide and hydrogen peroxide reactive species [31], which corroborates our data.

**Table 3:** DPPH^•^ radical scavenging, superoxide dismutase-*like* and catalase-*like* activities in different accessions of *Lippia alba*.

Accessions of *Lippia alba*	DPPH^•^ radical scavenging (IC_50_) ^#^	Superoxide dismutase-*like* activity (IC_50_) ^§^	Catalase-*like* activity (mmol of H_2_O_2_ decomposed/min)
Barra do Quaraí	0.20 ± 0.01 ^c^	20.05 ± 0.42 ^b^	23.75 × 10^2^ ± 193.65 ª
Caxias do Sul	0.23 ± 0.01 ^a^^,^^b^	26.71 ± 0.06 ^a^	19.37 × 10^2^ ± 153.09 ^e^
Harmonia	0.09 ± 0.01 ^f^	10.43 ± 0.50 ^d^	21.87 × 10^2^ ± 153.09 ^b^
Pelotas	0.23 ± 0.01 ^a^^,^^b^	13.41 ± 0.30 ^c^	15.53 × 10^2^ ± 141.74 ^g^
Pinheira	0.24 ± 0.01 ^a^	5.80 ± 0.12 ^e^	17.81 × 10^2^ ± 173.59 ^f^
Porto Alegre	0.14 ± 0.01 ^d^	13.89 ± 0.26 ^c^	19.68 × 10^2^ ± 187.50 ^d^
Santa Vitória do Palmar	0.12 ± 0.01 ^e^	14.27 ± 0.46 ^c^	21.75 × 10^2^ ± 167.71 ^c^

**^#^** Amount (%) of *Lippia alba* extract required to scavenge 50% of the 1,1-diphenyl 2-picrylhydrazyl radical. **^§^** µL of extract required to reduce 50% of the adrenochrome formation. Different letters correspond to values significantly different using an analysis of variance (ANOVA) and Tukey’s post hoc test for *p* ≤ 0.05 for each assay.

**Table 4:** Pearson correlations between phenolic compounds of *Lippia alba* accessions and DPPH^•^, superoxide dismutase-*like* and catalase-*like* assays.

	Total phenolic content	Apigenin	Luteolin	Naringin	Rutin
**DPPH^•^**	0.846 *	0.662 *	0.718 *	0.646 *	0.893 *
**SOD-*like***	0.771 *	0.884 *	0.409 *	0.353 **	0.700 *
**CAT-*like***	0.927 *	0.835 *	0.719 *	0.634 **	0.958 *

Statistically significant * for *p* ≤ 0.01 and ** for *p* ≤ 0.05.

### 3.3. Effects of *Lippia alba* Extract in Brain and Liver Tissue Homogenates

Santa Vitória do Palmar accession, which exhibited the highest naringin and total phenolic contents was also studied in tissue homogenates of Wistar rats. The cerebral cortex, cerebellum and hippocampus are the foremost integrative areas that are affected in various neurodegenerative disorders, such as amyotrophic lateral sclerosis, Alzheimer’s and Parkinson’s diseases [32,33]. Cerebral cortex and hippocampus are associated with cognition and feedback stress control, and the cerebellum controls motor function [34]. Free radicals are also associated with the occurrence of some liver diseases, primarily through lipid peroxidation [35].

**Table 5:** Lipid and protein damage in rat cerebellum, cerebral cortex, hippocampus and liver cells treated with *L. alba*.

Treatments	Lipid damage (nmol of TBARS/mg of protein)				Protein damage (nmol of DNPH/mg of protein)			
	Cerebellum	Cerebral Cortex	Hippocampus	Liver	Cerebellum	Cerebral Cortex	Hippocampus	Liver
Control	12.85 ± 0.90 ^b^	7.17 ± 0.41 ^c^	26.80 ± 1.67 ^c^	14.14 ± 1.26 ^c^	0.76 ± 0.01 ^c^	0.58 ± 0.01 ^c^	0.11 ± 0.01 ^b^	0.26 ± 0.02 ^b^
H_2_O_2_	24.05 ± 1.46 ^a^	43.73 ± 3.75 ^a^	76.32 ± 5.36 ^a^	36.75 ± 0.89 ^a^	2.06 ± 0.03 ^a^	1.63 ± 0.03 ^a^	0.18 ± 0.01 ^a^	0.64 ± 0.02 ^a^
*L. alba*	11.46 ± 0.99 ^b^^,c^	8.12 ± 0.57 ^b^^,c^	25.48 ± 1.92 ^c^	13.67 ± 0.73 ^c^	0.21 ± 0.02 ^d^	0.50 ± 0.03 ^d^	0.08 ± 0.01 ^c^	0.24 ± 0.01 ^b^
*L. alba* + H_2_O_2_	9.90 ± 0.89 ^c^	10.73 ± 0.27 ^b^	38.87 ± 0.98 ^b^	27.57 ± 1.18 ^b^	0.92 ± 0.03 ^b^	1.36 ± 0.01 ^b^	0.10 ± 0.01 ^b^^,c^	0.29 ± 0.01 ^b^

Tissues were incubated for 30 min in the presence of the *Lippia alba* extract (10 μg/mL) followed by 1 h in the presence of H_2_O_2_. Different letters correspond to values significantly different using analysis of variance (ANOVA) and Tukey’s post hoc test for *p* ≤ 0.05 for each tissue evaluated.

**Table 6:** Superoxide dismutase (SOD) and catalase (CAT) activities in rat cerebellum, cerebral cortex, hippocampus and liver cells treated with *L. alba*.

Treatments	SOD activity (U/mg of protein)				CAT activity (U/mg of protein)			
	Cerebellum	Cerebral Cortex	Hippocampus	Liver	Cerebellum	Cerebral Cortex	Hippocampus	Liver
Control	93.08 ± 1.75 ^b^	12.53 ± 0.24 ^b^	77.70 ± 1.26 ^b^	18.91 ± 0,93 ^b^	41.79 ± 2.40 ^b^	5.72 ± 0.54 ^b^	24.04 ± 2.17 ^b^	101.79 ± 7.58 ^b^
H_2_O_2_	105.37 ± 1.72 ^a^	64.30 ± 0.70 ^a^	122.33 ± 0.71 ^a^	53.01 ± 3.08 ^a^	60.37 ± 3.08 ^a^	26.74 ± 2.06 ^a^	58.33 ± 3.61 ^a^	144.64 ± 7.58 ^a^
*L. alba*	91.93 ± 2.42 ^b^	13.20 ± 0.20 ^b^	76.91 ± 1.33 ^b^	19.25 ± 1.41 ^b^	40.84 ± 3.08 ^b^	4.87 ± 0.46 ^b^	21.92 ± 2.08 ^b^	92.86 ± 6.19 ^b^
*L. alba* + H_2_O_2_	84.60 ± 3.75 ^b^	13.67 ± 0.18 ^b^	81.51 ± 6.25 ^b^	21.54 ± 0.33 ^b^	38.18 ± 2.17 ^b^	7.63 ± 0.69 ^b^	23.83 ± 2.26 ^b^	52.23 ± 3.79 ^c^

Tissues were incubated for 30 min in the presence of the *Lippia alba* extract (10 μg/mL) followed by 1 h in the presence of H_2_O_2_. Different letters correspond to values significantly different using analysis of variance (ANOVA) and Tukey’s post hoc test for *p* ≤ 0.05 for each tissue evaluated.

In the present work, the treatment with *L. alba* extract alone did not induce oxidative damage to lipids or proteins or modulate the activities of SOD and CAT enzymes in any of the assayed tissues (Table 5 and Table 6). On the other hand, *L. alba* pretreatments minimize H_2_O_2_-induced lipid (TBARS) and protein oxidative damage in CNS and liver tissues of Wistar rats (Table 5). TBARS assay is one of the oldest and most frequently used tests for the measurement of the peroxidation of fatty acids and membranes. Malondialdehyde is the main product of lipid peroxidation, and this product is a powerful genotoxic and carcinogenic compound [31]. Proteins are also targets of oxidative modification by reactive oxygen species. These reactions often lead to the modification of certain amino acid residues and the formation of carbonyl derivates, which are linked to losses in physiological functions under pathological processes or during aging [36]. Pretreatment with *L. alba* extracts prevented the H_2_O_2_-induced increase in SOD and CAT activities in CNS and liver tissues (Table 6). *L. alba* extracts contained high levels of phenolic compounds (Table 2), which seems to be correlated with the antioxidant effects observed in this study. Polyphenols may act as hydrogen donators and/or reducing agents [37], and exert neuroprotective [14,15,16] and hepatoprotective [16] effects. It is known that these compounds can play an important role in delaying the onset of age-related health disorders [38,39]. However, more research is needed to better understand the mechanisms through which polyphenols are able to act, mainly at physiological relevant levels. Therefore, the therapeutic potential of these natural compounds still remains to be confirmed in clinical conditions.

## 4. Conclusions

These data demonstrated that different *L. alba* accessions presented different phenolic profiles and antioxidant activities. It was showed, for the first time, that *L. alba* extracts possessed high levels of naringin and rutin, which are flavonoids with valuable biological properties. Therefore, the biological activities of the *L. alba* extracts is an important issue for further investigations in the ethnopharmacological sciences.
